# Pseudogap and proximity effect in the Bi_2_Te_3_/Fe_1+y_Te interfacial superconductor

**DOI:** 10.1038/srep32508

**Published:** 2016-09-02

**Authors:** M. Q. He, J. Y. Shen, A. P. Petrović, Q. L. He, H. C. Liu, Y. Zheng, C. H. Wong, Q. H. Chen, J. N. Wang, K. T. Law, I. K. Sou, R. Lortz

**Affiliations:** 1 Department of Physics, The Hong Kong University of Science and Technology, Clear Water Bay, Kowloon, Hong Kong S.A.R., China; 2 CorreLab, Gerbang Institute for Complex Matter, 81560 Nusajaya, Johor, Malaysia

## Abstract

In the interfacial superconductor Bi_2_Te_3_/Fe_1+y_Te, two dimensional superconductivity occurs in direct vicinity to the surface state of a topological insulator. If this state were to become involved in superconductivity, under certain conditions a topological superconducting state could be formed, which is of high interest due to the possibility of creating Majorana fermionic states. We report directional point-contact spectroscopy data on the novel Bi_2_Te_3_/Fe_1+y_Te interfacial superconductor for a Bi_2_Te_3_ thickness of 9 quintuple layers, bonded by van der Waals epitaxy to a Fe_1+y_Te film at an atomically sharp interface. Our data show highly unconventional superconductivity, which appears as complex as in the cuprate high temperature superconductors. A very large superconducting twin-gap structure is replaced by a pseudogap above ~12 K which persists up to 40 K. While the larger gap shows unconventional order parameter symmetry and is attributed to a thin FeTe layer in proximity to the interface, the smaller gap is associated with superconductivity induced via the proximity effect in the topological insulator Bi_2_Te_3_.

Tailoring a topological superconductor by combining the topologically protected surface states of a TI with a superconductor via the proximity effect is of enormous theoretical and technological interest, principally due to the possibility of finding the Majorana fermionic states which are predicted to exist in the vortex cores of topological superconductors^1^,^2^. A novel Bi_2_Te_3_/Fe_1+y_Te heterostructure^3^ may represent a promising material. Although both parent materials are non-superconducting, the interface becomes a 2D superconductor which undergoes a characteristic 2D Berezinski-Kosterlitz-Thouless (BKT) superconducting transition^4^,^5^,^6^. Bi_2_Te_3_ is a 3D topological insulator (TI) whose surface states consist of a single Dirac cone at the Γ point^7^, while Fe_1+y_Te is the parent compound of the ‘11’ family of Fe-based superconductors. Despite the exact mechanism for superconductivity in Bi_2_Te_3_/Fe_1+y_Te remaining unknown, it has been shown previously that any doping effect by O, Bi or Te impurities can be excluded^3^. Evidence that TI surface states play a role in the emergence of superconductivity is found in the fact that a critical thickness of the Bi_2_Te_3_ layer is required^3^. As the number of Bi_2_Te_3_ quintuple layers (QL) increases, *T*_c_ rises from ~1.2 K (1 QL) to 12 K (5 QL) and then saturates. This correlates with the 5 QL critical thickness required to form a fully-developed surface state in Bi_2_Te_3_^8^,^9^. The heterostructure may thus represent a model system to study proximity-induced topological superconductivity in the Bi_2_Te_3_ layer, and a highly complex superconducting mechanism is likely involved in the interfacial superconductivity.

In this article, we report directional point-contact data measured for current injection (a) parallel to the interface into the edge of the heterostructure, simultaneously probing both layers and (b) perpendicular to the interface into the top Bi_2_Te_3_ layer. We observe a pronounced twin gap structure with a large gap of unconventional pairing symmetry, corresponding to the interfacial superconductivity, and a smaller gap which opens below the characteristic BKT temperature and is associated with proximity-induced superconductivity in the topologically insulating Bi_2_Te_3_ layer. In addition, a pseudogap is observed which extends up to 40 K.

Point-contact spectroscopy is an energy-resolved technique directly probing the amplitude, symmetry and temperature-dependence of the superconducting gap^10^. Our data were acquired using bilayers of Bi_2_Te_3_(9QLs)/Fe_1+y_Te, chosen for their high *T*_c_ = 12 K. The Van-der-Waals bonding between the materials results in extremely high quality atomically-sharp interfaces.

Figure 1 displays the temperature-dependent resistance of the heterostructure, as measured with 4 contacts established by silver-loaded paint on top of the Bi_2_Te_3_ surface. An insulator-to-metal transition is visible in the form of a broad maximum at 76 K, which we associate with the antiferromagnetic transition of the bulk Fe_1+y_Te layer. At lower temperatures, the resistance passes through a minimum at 24 K before increasing steeply. The overall resistive behavior (including this increase) is typical for FeTe with a high content of interstitial excess Fe^11^, and can therefore be attributed to the bulk FeTe layer of our heterostructure. Indeed, high-resolution energy-dispersive X-ray spectroscopy in a scanning transmission electron microscope indicates that *y *= 0.15 ± 0.02 in our Fe_1+y_Te bulk layer. It should be noted that FeTe does not exist in stoichiometric form and always has some excess Fe in the form of individual interstitial ions^12^. Superconductivity in the vicinity of the Bi_2_Te_3_/Fe_1+y_Te interface reveals itself by a rapid resistance decrease below *T*_c_ = 12 K until zero resistance is reached at *T*_0_ = 8 K. From Fig. 1, the overall behavior of our heterostructure is in good agreement with our previous work^3^, in which we have shown that the broadness of the resistance drop associated with the superconducting transition between 12 K and 8 K is intrinsic, and the transition falls into the 2D-XY universality class of a BKT transition. It is important to note that the resistance is composed of 3 parallel components: the Bi_2_Te_3_ layer, the bulk Fe_1+y_Te layer and the intermediate thin interfacial layer. The resistance drop below 12 K is associated with the diverging superconducting correlation length in the interfacial layer upon approaching the BKT transition. However, 12 K is not necessarily the onset of the superconducting transition: the rapidly increasing resistance of the interface will cause the current to gradually move away from the interfacial region into the 140 nm thick bulk Fe_1+y_Te layer, which then begins to shunt the interface resistance. Therefore, the normal state resistance of the interface will be largely hidden due to the large Fe_1+y_Te thickness (140 nm) relative to that of the thin superconducting interfacial layer.

Figure 2a shows the temperature dependent point-contact spectra for the nano-contact on the heterostructure edge. At the highest temperatures a smooth parabolic background is seen, with little difference in data acquired at 70, 50 and 40 K. Below 40 K a pseudogap develops symmetrically around *V*_b_ = 0, gradually deepening as the temperature falls. Until 15 K, the gap is rounded at low energy, but at 12 K the conductance flattens around *V*_b_ = 0 prior to the emergence of a zero bias conductance peak (ZBCP) below ~10 K. Concurrently, shoulder-like structures develop at ~10 mV and ~5 mV, which as we will now demonstrate correspond to a phase-coherent superconducting twin-gap structure^13^.

To fit the temperature dependence of our data, we primarily employ a modified Blonder-Tinkham-Klapwijk (BTK) model for finite-transparency tunnel junctions^14^, excluding the energy range of the ZBCP. Our fits are based on a 1D BTK model for simplicity, since higher-dimensional models are equivalent to the 1D case except for small shifts in the barrier heights *Z*. In such a highly two-dimensional superconductor, fluctuations are expected to significantly reduce the quasiparticle lifetime; we account for this in our model with an energy-dependent Dynes parameter^15^ of the form *Γ*exp[(│*V*_b_│−Δ)/*W*] where *Γ* and *W* are free parameters. The shoulders at ~5 and ~10 mV are modeled by an anisotropic two-band s-wave order parameter Δ(*α*+(1 − *α*) cos *θ*)^16^, in which we determine the gap anisotropies α_1,2_ from a fit at our lowest achievable temperature 0.27 K, then fix *α*_1,2_ at these values for all other temperatures.

The fitting results are shown in Fig. 2b: we are able to accurately reproduce our experimental data – including the double-gap structure – across the entire energy range. Since our BTK fits indicate large barrier heights (*Z*_1_ ≥ 0.35 for Δ_1_ and *Z*_2_ ~ 1000 for Δ_2_), we also attempt to model our data using a two-band Dynes model (with a metallic conduction component to compensate for the lower barrier Z_1_). Both models yield similar results: at 0.27 K, Δ_1_ = 6 meV and Δ_2_ = 12 meV from the BTK model, while Δ_1_ = 4 meV and Δ_2_ = 13 meV from the Dynes fit. Δ_1_ exhibits a pronounced anisotropy α ~ 0.7, whereas Δ_2_ is approximately isotropic (α = 1). In each case, Δ_2_ provides the dominant contribution to the spectral weight: 60 ± 5% versus 40 ± 5% for Δ_1_ in the BTK model, compared with a Δ_1_:Δ_2_:metallic ratio of 8 ± 0.5%: 46 ± 4% : 46 ± 4% in the Dynes model. The smaller values for Δ_1_ and its spectral weight from the Dynes fit are due to non-negligible Andreev reflections, which cannot be perfectly simulated by the metallic component within this model. To account for the presence of the ZBCP, we also attempted to reproduce this data using a two-gap *d*-wave model, but no improvement of the fit or significant variation of the gap values were observed compared to the anisotropic *s*-wave case. Δ_1_ closes at 8 K while Δ_2_ appears to close at 40 K. The magnitude of Δ_2_ abruptly increases above 8 K. As we will demonstrate later, Δ_2_ actually consists of a large superconducting gap which transforms continuously into a pseudogap in the temperature range between 8 and 12 K. The pseudogap then closes at 40 K.

In Fig. 3 we present the differential conductance upon injecting the current through a scanning probe tip on the Bi_2_Te_3_ surface. The current was injected perpendicular to the film and hence only probes the Bi_2_Te_3_ layer. A small 5.4 meV gap dominates the conductance at low temperature, in excellent agreement with the small gap Δ_1_ observed upon injecting the current into the edge of the interface. The conductance saturates for *V*_b_ > 6 mV and no signature of the larger gap Δ_2_ is observed. This suggests that the Bi_2_Te_3_ layer becomes superconducting and is responsible for Δ_1_, similar to what has been observed in Bi_2_Sr_2_CaCu_2_O_8+δ_/Bi_2_Se_3_^17^,^18^, Bi_2_Sr_2_CaCu_2_O_8+δ_/Bi_2_Te_3_^18^ or Bi_2_Sr_2_CaCu_2_O_8+δ_/Bi_2_Te_2_Se^19^ junctions grown on a cuprate high-temperature superconductor, or in proximity contact with classical superconductors^20^. Here it should be noted that certain other groups have reported the absence of a proximity effect in Bi_2_Sr_2_CaCu_2_O_8+δ_/Bi_2_Se_3_ junctions^21^,^22^. Additional point contact data can be found in the supplementary information.

In Fig. 4 we plot the point-contact spectra in 0 and 15 T applied (a) perpendicular and (b) parallel (field perpendicular to the current injection direction) to the film plane. Note that for technical reasons, different edge contacts on the same sample have been used, and the spectra in Fig. 4b are somewhat broader but show qualitatively the same features. The gap structure and ZBCP are quite robust with respect to magnetic fields, irrespective of their orientation. The spectra hardly change in 15 T: the gap becomes marginally shallower, but the gap energies Δ_1,2_ do not shrink significantly. This resilience of the overall gap structure demonstrates that the Cooper pairing strength is almost impervious to strong fields, in direct contrast with the critical field *H*_c2_ = 17 T which has been estimated from resistance data^3^. We deduce that *H*_c2_ merely corresponds to a field-induced loss of phase coherence. We observe reductions in the ZBCP height of 20% and 30% in 15 T applied in-plane and perpendicular to the interface, respectively, measured with respect to the minimum in the d*I*/d*V* curve. The ZBCP does not experience any splitting, regardless of the magnetic field orientation. In Fig. 4c we show the effect of a 9T magnetic field on the pseudogap: a magnetic field has a weak suppressing effect at temperatures up to 40 K.

The large size of Δ_2_ ≥ 12 meV is a striking feature of our Bi_2_Te_3_/Fe_1+y_Te heterostructure. It exceeds the superconducting gap in bulk FeSe^23^ or FeSe_1−x_Te_x_^24^ by at least a factor of 4, despite the *T*_c_ of our heterostructures being comparable to *T*_c_ in these bulk materials. Our data constitute a demonstration of the potential for strong-coupling superconductivity which could persist up to far higher temperatures than the critical temperatures observed in the bulk iron-chalcogenides. The origin of this gap enhancement is unclear, but since the presence of the Bi_2_Te_3_ layer is essential for the appearance of superconductivity^3^, it is possible that the topological surface states of Bi_2_Te_3_ play a certain role.

Multiband superconductivity with various gaps has been reported in various Fe-based superconductors^10^,^25^,^26^ and is usually attributed to multiple electronic bands crossing the Fermi level in the same material. However, as we are probing the properties of the FeTe and Bi_2_Te_3_ layers in parallel, the twin-gap feature could also originate from two spatially separated regions each with its corresponding electronic bands involved. Superconductivity induced by the proximity effect in the Bi_2_Te_3_ layer could therefore play a role in the opening of the second gap. A proximity-induced small gap has previously been observed in devices of Bi_2_Se_3_ and Bi_2_Te_3_ which were mechanically bonded to the cuprate high-*T*_c_ Bi_2_Sr_2_CaCu_2_O_8+δ_: these showed a similar reduction of the gap in the cuprate when the TI became superconducting^18^.

The temperature dependence of the gaps extracted from the BTK and Dynes fits is shown in Fig. 2c,d: it is clear that both models yield qualitatively identical results. Below 2 K the smaller gap Δ_1_ is approximately constant; upon increasing the temperature its magnitude decreases rapidly, reaching zero at ~8 K. In contrast, the larger gap Δ_2_ is almost temperature-independent between 10 K and 25 K, gradually decreasing towards zero when the temperature is increased further up to 40 K. Between 10 K and 8 K, Δ_2_ is slightly reduced, which clearly correlates with the opening of Δ_1_. Below 8 K, the sample exhibits zero resistance^3^ and is hence globally phase-coherent. Although the pronounced coherence peaks, which are characteristic of superconducting tunneling spectra, are absent from our data due to the short quasiparticle lifetimes imposed by low-dimensional fluctuations, we may nevertheless infer the presence of coherence by the sharp falls in *dI/dV* close to Δ_1,2_. Above 8 K, global phase coherence is lost, the resistance gradually increases until the normal state is reached at 12 K and Δ_1_ vanishes, while Δ_2_ becomes a pseudogap which persists up to 40 K. The Dynes parameter Γ_2_ describing the quasiparticle lifetime in Δ_2_ also rises steeply above 8 K (Fig. 2e), supporting our observed loss of phase coherence above this temperature.

The edge contact data does not allow us to judge whether Δ_2_ closes at 8 K and is replaced by a pseudogap due to a competing order, or if it transforms continuously into the high-temperature pseudogap, thus suggesting a phase-incoherent superconducting (i.e. pairing) origin. A pronounced pseudogap state above the superconducting critical temperature is most famous in the cuprate high temperature superconductors^27^, and its origin is still widely debated. In addition, various Fe-based superconductors display signatures of a pseudogap well above *T*_c_^10^,^24^,^28^,^29^,^30^,^31^ and its origin has been suggested to be due to fluctuations of a nematic electronic order^31^,^32^,^33^.

The pseudogap opens well below the antiferromagnetic transition at ~80 K of the bulk Fe_1+y_Te layer as seen in the resistivity (Fig. 1)^34^ and thus does not appear to be related to its magnetic ordering. Similar to Se-doped FeTe, it is likely that the magnetic order in the interface region is weakened by charge transfer across the interface from the *n*-doped Bi_2_Te_3_ layer. In addition, the topological surface state may contribute electrons with a strong spin-orbit coupling, thus further suppressing the interfacial magnetic order and paving the way for the emergence of superconductivity. In Ba_0.85_K_0.15_Fe_2_As_2_ it has been shown by angle resolved photoemission spectroscopy that the SDW order is associated with a ~20 meV gap which forms below the SDW ordering temperature^35^. At low temperature the SDW gap is reduced concurrently with the onset of superconducting order, in a very similar manner to what we observe for the larger gap Δ_2_ above 8 K in Fig. 2c,d. A normal state origin is thus compatible with the increase of Δ_2_ above 8 K, which is suggestive of a replacement of the superconducting gap by a normal state pseudogap. This suggests that the pseudogap which we observe has a competitive relationship with superconductivity and is presumably related to SDW and/or nematic order, which are therefore likely to develop below ~40 K in the interface region.

On the other hand, we have previously shown that the interfacial superconductivity in our heterostructures lies in the extreme 2D limit and the resistance drop and *IV* characteristics can be perfectly modelled by a BKT transition^3^. This represents a pure 2D phase-ordering transition of Cooper pairs which are already formed at higher temperature, where the phase of the superconducting order parameter is stabilized below a characteristic temperature *T*_BKT_ (lying just above *T*_0_), at which thermally-induced vortices and anti-vortices are bound into pairs^4^,^5^,^6^. This naturally implies the existence of phase-incoherent Cooper pairs within a certain temperature range above *T*_BKT_, creating a pseudogap in the density of states. The 10 meV magnitude of this superconducting gap indicates a potential for strong coupling superconductivity with stable Cooper pairing at temperatures well above 12 K. Superconducting fluctuations have been observed at temperatures many times higher than *T*_c_ in strongly-underdoped layered cuprate HTSCs^36^,^37^, where they contribute in part to the pseudogap formation. Furthermore, scanning tunneling spectroscopy on ultrathin titanium nitride films has shown that the strong phase fluctuations associated with two-dimensionality can induce a pseudogap in conventional superconducting films at temperatures up to 14 times *T*_c_^38^. A superconducting origin for the pseudogap in the interfacial superconductor Bi_2_Te_3_(9QLs)/Fe_1+y_Te is supported by the fact that the pseudogap is partially suppressed by a magnetic field of 9 T in temperatures up to 40 K (Fig. 4c), which could be a consequence of pair-breaking effects and is not expected for the SDW gap in an Fe based superconductor. If the pseudogap had an entirely phase-incoherent superconducting origin, then the reduction of the gap upon transformation into a real superconducting gap Δ_2_below ~8 K could be caused by the opening of the proximity-induced gap Δ_1_ in the Bi_2_Te_3_ layer, similar to the reduction of the superconducting gap observed in Bi_2_Sr_2_CaCu_2_O_8+δ_ in proximity contact to a TI^18^.

However, in a pseudogap which is caused entirely by fluctuations of the superconducting order parameter, it is expected that the zero-bias conductance G_N_ should vary linearly with ln(ln(*T*/*T*_0_))^38^,^39^, where *T*_0_ is the phase coherence temperature, represented by the establishment of zero resistivity at 8 K. From Fig. 5 this trend is not observed in our data, thus suggesting that a competing normal-state pseudogap at least partially contributes to the spectra. The most likely explanation of the pseudogap is thus a mixture of a normal state pseudogap (e.g. caused by interfacial SDW or nematic order) and a phase incoherent superconducting pseudogap caused by the strong phase fluctuations of a 2D superconductor.

As demonstrated by our point contact data on the top of the Bi_2_Te_3_ layer, interfacial contact with the Fe_1+y_Te induces superconductivity in Bi_2_Te_3_, which is therefore a potential candidate to host a topological superconducting state^40^. The combination of superconductivity with the non-trivial topological symmetry of the surface states in a TI naturally evokes the question whether the ZBCP could be caused by Majorana bound states^41^,^42^,^43^. Care has been taken to eliminate any spurious origin for the ZBCP, e.g. heating or proximity effects^44^: (1) our high-resistance point-contact lies comfortably within the ballistic spectroscopic tunneling regime, (2) the spectra were verified to be identical upon increasing and decreasing the tunnel current and (3) the ZBCP width remains roughly constant at all temperatures below 8 K (Fig. 2a), despite the gap energy Δ_1_ increasing from zero to 5 meV within this temperature range. The ZBCP is therefore of intrinsic origin. The proximity effect of an *s*-wave superconductor on a TI^1^ creates a fully gapped energy spectrum without any in-gap states; in this case, Majorana modes would only be created in the vortex cores in an applied magnetic field. Since the pairing symmetry in Bi_2_Te_3_(9QLs)/Fe_1+y_Te remains unclear, two possible mechanisms exist for the formation of Majorana edge states. One possibility is that the contact with FeTe drives the Bi_2_Te_3_ layer to become an intrinsic topological superconductor with Majorana surface states^40^,^41^,^45^,^46^, e.g. by a charge transfer effect, instead of a proximity-induced superconductor. This would explain the ZBCP observed in the point contact spectra of both the edge (Fig. 2) and the top contacts (Fig. 3). In a magnetic field the ZBCP should be suppressed due to broken time-reversal symmetry: a reduction in the ZBCP height is indeed observed in high magnetic fields, but the effect is only ~20% in 14T. This could be a consequence of a particularly strong Rashba spin-orbit coupling at the interface as well as the extremely high critical field for pairing. A detailed theoretical study of the possible pairing symmetries in Bi_2_Te_3_ would be required to confirm this possibility. The alternative mechanism requires nodal *d*_*x*_^2^_−__*y*_^2^ superconductivity (associated with the Fe_1+y_Te) combined with Rashba spin-orbit coupling (enhanced by the topological surface states of Bi_2_Te_3_)^47^. Although a *d*-wave order parameter alone could create a fermionic ZBCP at the sample edge, a ZBCP composed of the fermionic edge states and Majorana fermions should form in the presence of strong spin-orbit coupling at zero field. It has been predicted that an in-plane applied field will split and shift the fermionic states to finite energy via the Zeeman effect, while the Majorana state remains at zero energy^47^. However, no splitting of the ZBCP is observed up to 15 T for applied fields parallel or perpendicular to the interface (Fig. 4a,b), and its height is rather small, thus rendering such a *d*-wave scenario unlikely. Furthermore, the ZBCP visible in our top contact (Fig. 3) is at odds with a *d*-wave scenario.

A more conventional explanation for the ZBCP is related to the large Fe excess in the Fe_1+y_Te layer: scanning probe measurements have recently shown that excess iron in Fe(Te,Se) superconductors causes pronounced local in-gap states, which do not split, shift or vanish in applied magnetic fields^48^. However, the presence of a ZBCP when tunneling directly into the Bi_2_Te_3_ layer (in which Fe impurities are absent) discourages this interpretation and rather points to an unconventional pairing mechanism as its origin.

A similar ZBCP was observed in Bi2212/Bi_2_Te_2_Se junctions with a very similar temperature and magnetic field dependence as in our heterostructure^19^. This suggests that such a ZBCP could be an universal feature of interfaces between unconventional superconductors and topological insulators. Its origin remains a mystery and requires further experiments such as angle resolved photoemission to clarify the exact electronic density of states in Bi_2_Te_3_/Fe_1+y_Te heterostructures.

Our Bi_2_Te_3_(9QLs)/Fe_1+y_Te heterostructures reveal a highly unusual superconducting state with an extraordinarily large superconducting gap, a pronounced pseudogap and compelling evidence for proximity-induced superconductivity in the topological insulating Bi_2_Te_3_ top layer. Our experiments alone are not able to prove the topological nature of this superconductivity, nor provide an indisputable explanation of the origin of the interfacial superconductivity. Nevertheless, it is clear that Bi_2_Te_3_(9QLs)/Fe_1+y_Te interfaces display a similarly rich behavior to the cuprate superconductors, which remain one of the major unsolved mysteries in physics.

## Methods

### Film growth

The heterostructure studied in this work was synthesized by a VG-V80H MBE system. A 50 nm ZnSe buffer was first grown on a GaAs(100) semi-insulating substrate. A 140 nm thick FeTe layer is then deposited on the buffer layer, followed by a 9QL thick Bi_2_Te_3_ film. Detailed information about the quality and characterization of this interface, including scanning transmission microscope micrographs and resistivity data may be found in Ref. 3. Our data were acquired using bilayers of Bi_2_Te_3_(9QLs)/Fe_1+y_Te, chosen for their high *T*_c_ = 12 K.

### Device fabrication and point contact spectroscopy

**Figure 6:**
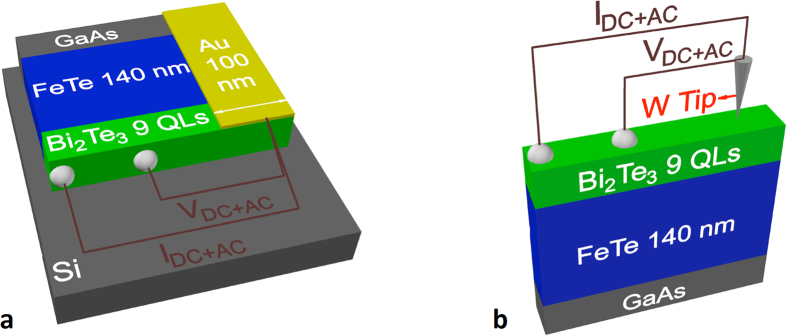
Schematic model of the point contact devices. (**a)** For the fabrication of a point contact at the edge of the Bi_2_Te_3_(9QLs)/Fe_1+y_Te heterostructure, a thin polished slab was glued onto a substrate with its edge facing upwards. A point contact was fabricated in form of a narrow 100 nm wide gold strip over the edge. An AC current (*I*_AC_) superimposed on a DC current (*I*_DC_) was injected from the strip to an ordinary highly conducting contact on the Bi_2_Te_3_ surface, while the resulting AC and DC voltages were measured between the strip and a second low-Ohmic contact by a lock-in amplifier and a DC voltmeter, respectively. (**b**) The point contact on the top Bi_2_Te_3_ surface was established by gently pressing a W tip against the surface. The measurements were then conducted in the same manner as described above for the edge contact.

To fabricate a point-contact device on the edge of the bilayer, a thin slab was attached to a silicon substrate with one edge facing upwards. Ordinary low-Ohmic contacts were prepared by RS 186–3593 silver conductive paint on the Bi_2_Te_3_ surface. The edge of the sample was finely polished, instantly covered by a thin layer of Au, and an isolated 100 nm wide Au strip was separated using a focused-ion-beam. The maximum contact area is ~100 × 149 nm^2^ (from the width of the Au strip and the total thickness of the FeTe/Bi_2_Te_3_ bilayer, respectively). A schematic drawing of the contact configuration is shown in Fig. 6. In order to reduce the effect of Ag pollution at the interface through our surface electrical contacts, we prepared all surface electrical contacts at a distance of at least 1 mm from the point contact junctions.

The edge contacts typically had resistances in the kΩ range and the data reproducibility was verified on 4 different devices. The point-contact spectral shape is strongly dependent on the tunnel barrier height parameter *Z*^10^, which is linked to the contact resistance. *Z* = 0 corresponds to pure Andreev reflection, while larger values represent the spectroscopic tunneling regime. In our contacts we consistently achieve Z ≥ 0.35 and our nanoscale contact area ensures that our experimental tunneling regime is ballistic and not thermal or diffusive, i.e. the applied bias voltage *V*_b_ corresponds to the electron injection energy. This is confirmed by the temperature-independent value of the normal-state contact resistance. A point contact with similar properties was established on the Bi_2_Te_3_ surface for perpendicular current injection with the help of a scanning probe device, by gently approaching a tungsten tip to the Bi_2_Te_3_ surface. The differential conductance *dI*/*dV* vs *V*_b_ was measured at temperatures from 0.27 K to 70 K in magnetic fields up to 15 T with a quasi-four-probe method, using a Keithley 6221 AC/DC current source to generate a small, constant-amplitude (10 nA) AC current *I*_*AC*_ with frequency 5 Hz, superposed on a ramped DC bias current. A standard lock-in technique in combination with a DC multimeter was used to measure *dI/dV* and *V*_b_ = *V*_*DC*_ across the junction.

## Additional Information

**How to cite this article**: He, M. Q. *et al*. Pseudogap and proximity effect in the Bi_2_Te_3_/Fe_1+y_Te interfacial superconductor. *Sci. Rep.* **6**, 32508; doi: 10.1038/srep32508 (2016).

## Supplementary Material

Supplementary Information

## Figures and Tables

**Figure 1:**
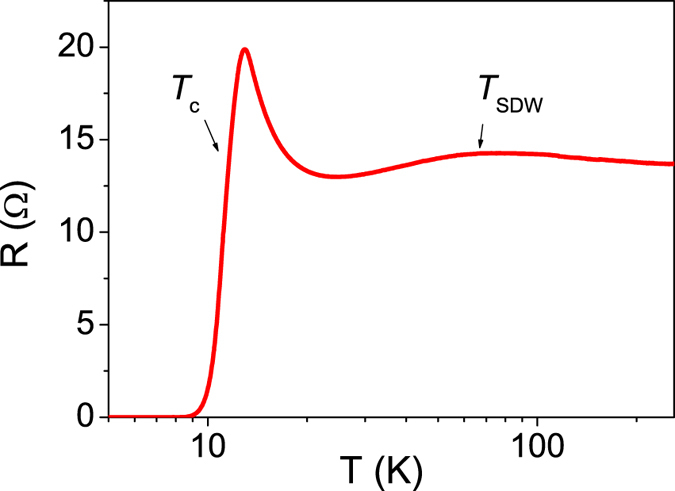
Temperature dependence of the electrical resistance of the device. The drop below 12 K is associated with the superconducting BKT transition of the interface region^3^, while the resistance above ~12 K is dominated by the bulk Fe_1+y_Te layer. *T*_SDW_ is the antiferromagnetic spin density wave ordering temperature of the bulk FeTe layer^12^.

**Figure 2:**
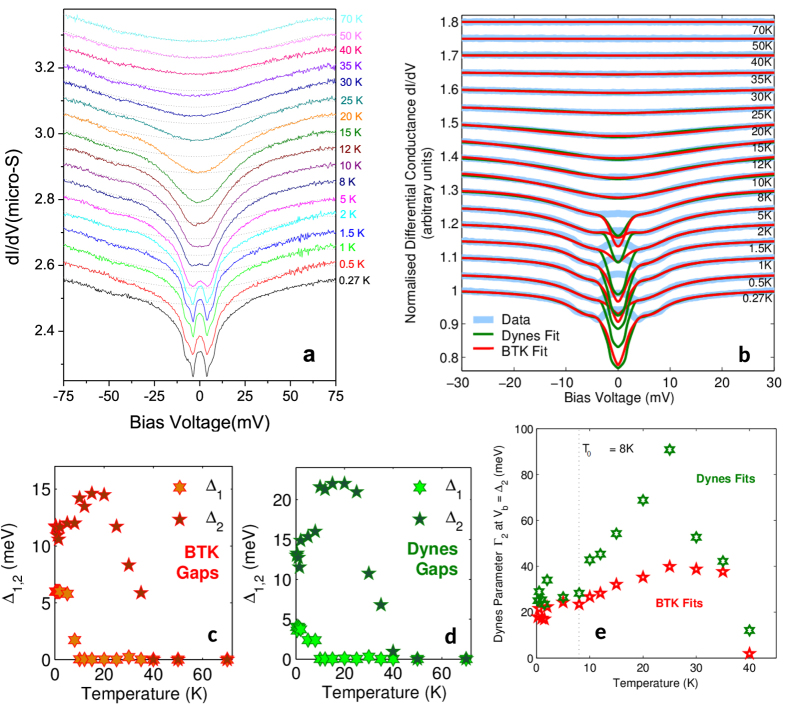
(**a**) Temperature-dependent differential conductance spectroscopy. The data was taken on a point-contact on the edge of the Bi_2_Te_3_(9QLs)/Fe_1+y_Te heterostructure. Offsets have been added for clarity. The dotted lines illustrate the normal-state background. (**b**) Data from 2(**a**) normalized to a polynomial background from the 70 K spectrum, together with fits using superconducting 2-gap BTK and Dynes models. Offsets have been added for clarity. The low voltage range containing the ZBCP (±3.5 mV) was excluded from the fit. (**c**,**d**) Temperature dependence of the two superconducting gaps Δ_1,2_ determined from BTK and Dynes models. (**e**) The energy dependent Dynes parameter Γ_2_ describing the quasiparticle lifetime in Δ_2_ obtained from the fits to the 2-gap Dynes and BTK models.

**Figure 3:**
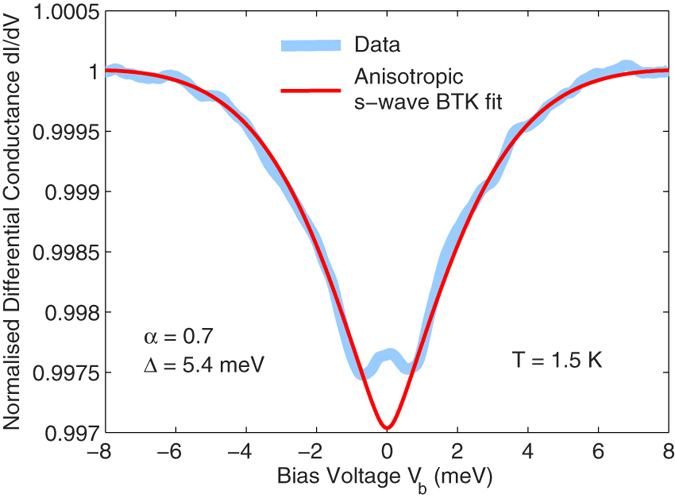
Zero-field conductance of a point contact on the Bi_2_Te_3_ surface. The same Bi_2_Te_3_(9QLs)/Fe_1+y_Te heterostructure was used as in Fig. 2. Data were acquired by injecting the current perpendicular to the film plane through a tungsten scanning probe tip in contact with the surface. A fit using an anisotropic *s*-wave single-gap BTK model is also shown: α = 0.7 and Δ = 5.4 meV, thus corroborating our results for Δ_1_ from our edge-contacted heterostructures.

**Figure 4:**
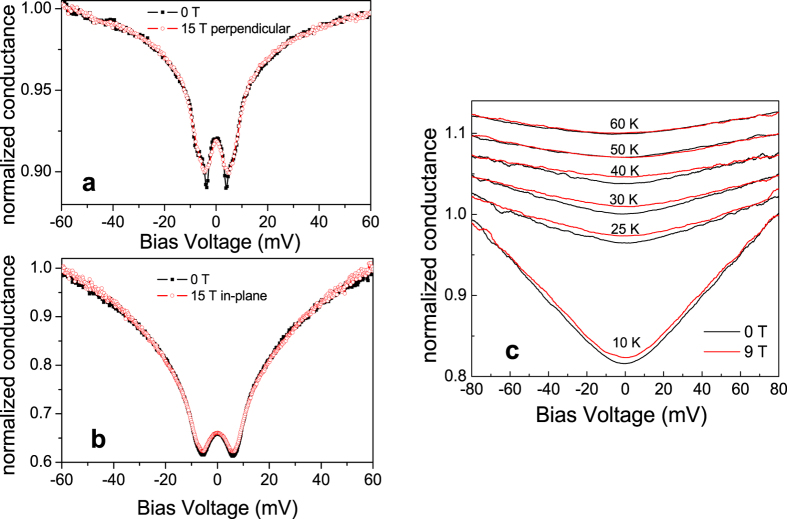
Differential conductance of the edge contact in magnetic fields. (**a,b**) Differential conductance of the edge contact in 0 T and 15 T at 0.27 K applied perpendicular (**a**) and parallel (**b**) to the interface. The spectra in (**b**) have been obtained on a different edge contact on the same sample, which show slightly broader spectra than at the contact in (**a**). (**c**) Pseudogap in the normalized spectra in 0 T and 9 T, illustrating a partial suppression of the pseudogap by the applied field at temperatures below 40 K.

**Figure 5:**
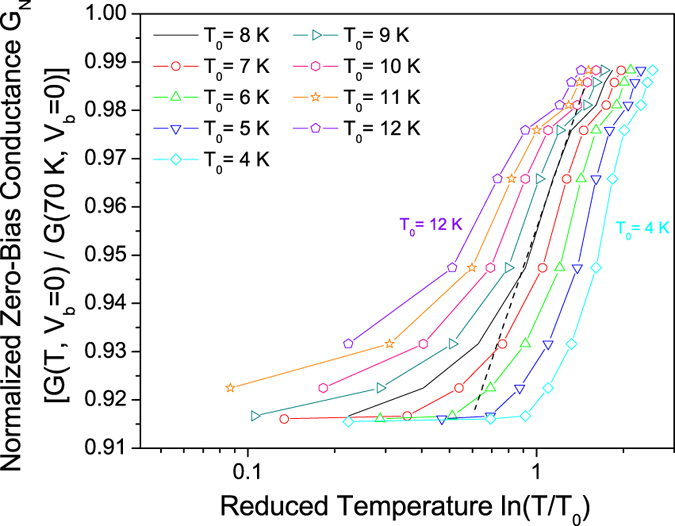
Temperature dependence of the zero-bias conductance. The graph shows temperature dependence of the normalized zero-bias conductance G_N_ above the phase-coherence temperature *T*_0_ as a function of the reduced temperature *ε* = ln(*T*/*T*_0_) for various choices of *T*_0_ surrounding our experimentally-determined *T*_0_ = 8 K (the dotted line is a guide to the eye). The absence of linear behavior over a sufficiently large range of *ε* suggests a competitive normal state contribution to the pseudogap, which likely competes with phase-incoherent pairing^38^,^39^.

## References

[b1] Fu L. & Kane C. L. Superconducting proximity effect and majorana fermions at the surface of a topological insulator. Phys. Rev. Lett. 100, 096407 (2008). 18352737 10.1103/PhysRevLett.100.096407

[b2] Xu J.-P. . Experimental detection of a Majorana mode in the core of a magnetic vortex inside a topological insulator-superconductor Bi_2_Te_3_/NbSe_2_ heterostructure, Phys. Rev. Lett. 114, 017001 (2015). 25615497 10.1103/PhysRevLett.114.017001

[b3] He Q. L. . Two-dimensional superconductivity at the interface of a Bi_2_Te_3_/FeTe heterostructure. Nat. Commun. 5 4247 (2014). 24953963 10.1038/ncomms5247

[b4] Kosterlitz J. M. & Thouless D. M. Ordering, metastability and phase transitions in two-dimensional systems. *J. Phys.* **C** 6, 1181–1203 (1973). 10.1088/0953-8984/28/48/481001 27665689

[b5] Kosterlitz J. M. The critical properties of the two-dimensional XY model. J. Phys. **C** 7, 1046–1060 (1974).

[b6] Berezinskii V. L. Destruction of long-range order in one-dimensional and two-dimensional systems having a continuous symmetry group I. classical systems. Sov. Phys. JETP 32, 493–500 (1971).

[b7] Chen Y. L. . Experimental realization of a three-dimensional topological insulator, Bi_2_Te_3_. Science 325, 178–181 (2009). 19520912 10.1126/science.1173034

[b8] Li Y.-Y. . Intrinsic topological insulator Bi_2_Te_3_ thin films on Si and their thickness limit. Adv. Mater. 22, 4002–4007 (2010). 20648518 10.1002/adma.201000368

[b9] Taskin A. A., Sasaki S., Segawa K. & Ando Y. Manifestation of topological protection in transport properties of epitaxial Bi_2_Se_3_ thin films. Phys. Rev. Lett. 109, 066803 (2012). 23006293 10.1103/PhysRevLett.109.066803

[b10] Daghero D., Tortello M., Ummarino G. A. & Gonnelli R. S. Directional point-contact Andreev-reflection spectroscopy of Fe-based superconductors: Fermi surface topology, gap symmetry, and electron–boson interaction. Rep. Prog. Phys. 74, 124509 (2011).

[b11] Koz C., Rößler S., Tsirlin A. A., Wirth S. & Schwarz U. Low-temperature phase diagram of Fe_1+y_Te studied using x-ray diffraction. Phys. Rev. B 88, 094509 (2013).

[b12] Bao W. . Tunable (δπ, δπ)-Type Antiferromagnetic Order in α-Fe(Te,Se) Superconductors. Phys. Rev. Lett. 102, 247001 (2009). 19659037 10.1103/PhysRevLett.102.247001

[b13] Eskildsen M. R. .Vortex imaging in magnesium diboride with H⊥c. Phys. Rev. B 68, 100508 (2003).

[b14] Blonder G. E., Tinkham M. & Klapwijk T. M. Transition from metallic to tunneling regimes in superconducting microconstrictions: excess current, charge imbalance, and supercurrent conversion. Phys. Rev. B 25, 4515 (1982).

[b15] Dynes C., Naraynamurti V. & Garno J. P. Direct measurement of quasiparticle-lifetime broadening in a strong-coupled superconductor. Phys. Rev. Lett. 41, 1509 (1978).

[b16] Petrović A. P. . Multiband superconductivity in the Chevrel phases SnMo_6_S_8_ and PbMo_6_S_8_. Phys. Rev. Lett. 106, 017003 (2011). 21231768 10.1103/PhysRevLett.106.017003

[b17] Wang E. . Fully gapped topological surface states in Bi_2_Se_3_ films induced by a d-wave high-temperature superconductor. Nat. Phys. 9, 621–625 (2013).

[b18] Zareapour P. . Proximity-induced high-temperature superconductivity in the topological insulators Bi_2_Se_3_ and Bi_2_Te_3_. Nat. Commun. 3, 1056 (2012). 22968702 10.1038/ncomms2042

[b19] Zareapour P. . Evidence for a new excitation at the interface between a high-T_c_ superconductor, *and a topological insulator*, Rev. B 90, 241106 (R) (2014).

[b20] Zhang D. . Superconducting proximity effect and possible evidence for Pearl vortices in a candidate topological insulator. Phys. Rev. B 84, 165120 (2011).

[b21] Yilmaz T. . Absence of a proximity effect for a thin-films of a Bi_2_Se_3_ topological insulator grown on top of a Bi_2_Sr_2_CaCu_2_O_8+δ_ cuprate superconductor. Phys. Rev. Lett. 113, 067003 (2014). 25148345 10.1103/PhysRevLett.113.067003

[b22] Xu S.-Y. . Fermi-level electronic structure of a topological-insulator/cuprate-superconductor based heterostructure in the superconducting proximity effect regime. Phys. Rev. B 90, 085128 (2014).

[b23] Song C.-L. . Suppression of superconductivity by twin boundaries in FeSe. Phys. Rev. Lett. 109, 137004 (2012). 23030114 10.1103/PhysRevLett.109.137004

[b24] Kato T. . Local density of states and superconducting gap in the iron chalcogenide superconductor Fe_1+δ_Se_1−x_Te_x_ observed by scanning tunneling spectroscopy. Phys. Rev. B 80, 180507 (2009).

[b25] Hardy F. . Calorimetric evidence of multiband superconductivity in Ba(Fe_0.925_Co_0.075_)_2_As_2_ single crystals. Phys. Rev. B 81, 060501 (R) (2010).

[b26] Tortello M. . Multigap superconductivity and strong electron-boson coupling in Fe-based superconductors: a point-contact Andreev-reflection study of Ba(Fe_1−x_Co_x_)_2_As_2_ single crystals. Phys. Rev. Lett. 105, 237002 (2010). 21231497 10.1103/PhysRevLett.105.237002

[b27] Timusk T. & Statt B. The pseudogap in high-temperature superconductors: an experimental survey. Rep. Prog. Phys. 62, 61–122 (1999).

[b28] Xu Y.-M. . Fermi surface dichotomy of the superconducting gap and pseudogap in underdoped pnictides. *Nat. Commun*. 2, 392 (2011). 10.1038/ncomms1394 21750547

[b29] Shimojima T. . Pseudogap formation above the superconducting dome in iron pnictides. Phys. Rev. B 89, 045101 (2014).

[b30] Kwon Y. S. . Evidence of a pseudogap for superconducting iron-pnictide Ba_0.6_K_0.4_Fe_2_As_2_ single crystals from optical conductivity measurements. New J. Phys. 14, 063009 (2012).

[b31] Arham H. Z. . Detection of orbital fluctuations above the structural transition temperature in the iron pnictides and chalcogenides. Phys. Rev. B 85, 214515 (2012).

[b32] Chu J.-H. . In-plane resistivity anisotropy in an underdoped iron arsenide superconductor. Science 329, 824–826 (2010). 20705856 10.1126/science.1190482

[b33] Harriger L. W. . Nematic spin fluid in the tetragonal phase of BaFe_2_As_2_. Phys. Rev. B 84, 054544 (2011).

[b34] Karki A. B. . Interplay between superconductivity and magnetism in Fe_1−x_Pd_x_Te. PNAS 110, 9283–9288 (2013). 23690601 10.1073/pnas.1307113110 PMC3677506

[b35] Yi M. . Dynamic competition between spin-density wave order and superconductivity in underdoped Ba_1−x_K_x_Fe_2_As_2_. Nat. Commun. 5, 3711 (2012). 10.1038/ncomms4711 24762657

[b36] Meingast C. . Phase fluctuations and the pseudogap in YBa_2_Cu_3_O_x_. Phys. Rev. Lett. 86, 1606 (2001). 11290204 10.1103/PhysRevLett.86.1606

[b37] Wang Y. . The onset of the vortex-like Nernst signal above T_c_ in La_2−x_Sr_x_CuO_4_ and Bi_2_Sr_2−y_La_y_CuO_6_. Phys. Rev. B 64, 224519 (2001).

[b38] Sacépé B. . Pseudogap in a thin film of a conventional superconductor. *Nat. Commun*. 1, 140 (2010). 21266990 10.1038/ncomms1140

[b39] Varlamov A. A. & Dorin V. V. Fluctuation resistance of Josephson junction, *Soviet Phys*. JETP 57, 1089–1096 (1983).

[b40] Schnyder A. P., Ryu S., Furusaki A. & Ludwig A. W. W. Classification of topological insulators and superconductors in three spatial dimensions. *Phys. Rev.* B 78, 195125 (2008).

[b41] Sasaki S. . Topological Superconductivity in Cu_x_Bi_2_Se_3_. Phys. Rev. Lett. 107, 217001 (2011). 22181913 10.1103/PhysRevLett.107.217001

[b42] Alicea J. New directions in the pursuit of Majorana fermions in solid state systems. Rep. Prog. Phys. 75, 076501 (2012). 22790778 10.1088/0034-4885/75/7/076501

[b43] Beenakker C. W. J. Search for Majorana fermions in superconductors. Annu. Rev. Con. Mat. Phys. 4, 113–136 (2013).

[b44] Sheet G., Mukhopadhyay S. & Raychaudhuri P. Role of critical current on the point-contact Andreev reflection spectra between a normal metal and a superconductor. Phys. Rev. B 69, 134507 (2004).

[b45] Fu L. & Berg E. Odd-parity topological superconductors: theory and application to Cu_x_Bi_2_Se_3_. Phys. Rev. Lett. 105, 097001 (2010). 20868184 10.1103/PhysRevLett.105.097001

[b46] Hsieh T. H. & Fu L. Majorana fermions and exotic surface Andreev bound states in topological superconductors: application to Cu_x_Bi_2_Se_3_. Phys. Rev. Lett. 108, 107005 (2012). 22463445 10.1103/PhysRevLett.108.107005

[b47] Yuan N. F. Q., Wong C. L. M. & Law K. T. Probing Majorana flat bands in nodal d_x_^2^_−y_^2^-wave superconductors with Rashba spin–orbit coupling. Physica E 55, 30–36 (2014).

[b48] Yin J.-X. . Observation of a robust zero-energy bound state in iron based superconductor Fe(Te,Se). Nat. Phys. 11, 543–546 (2015).

