# Supplementary material for: Pseudogap and proximity effect in the Bi2Te3/Fe1+yTe interfacial superconductor
Source: Sci Rep. 2016 Sep 2;6:32508. doi: 10.1038/srep32508 (PMC5009436; doi:10.1038/srep32508)
Supplement: Supplementary Information [file srep32508-s1.pdf]

Pseudogap and proximity effect in the $Bi_2Te_3/Fe_{1+y}Te$ interfacial superconductor

M. Q. He[1], J. Y. Shen[1], A. P. Petrovic[2], Q. L. He[1], H. C. Liu[1], Y. Zheng[1], C. H. Wong[1], Q. H. Chen[1], J. N. Wang[1], K. T. Law[1], I. K. Sou[1] and R. Lortz[1]

[1]Department of Physics, The Hong Kong University of Science and Technology, Clear Water Bay, Kowloon, Hong Kong S. A. R., China.

[2]CorreLab, Gerbang Institute for Complex Matter, 81560 Nusajaya, Johor, Malaysia

Supplementary Figures

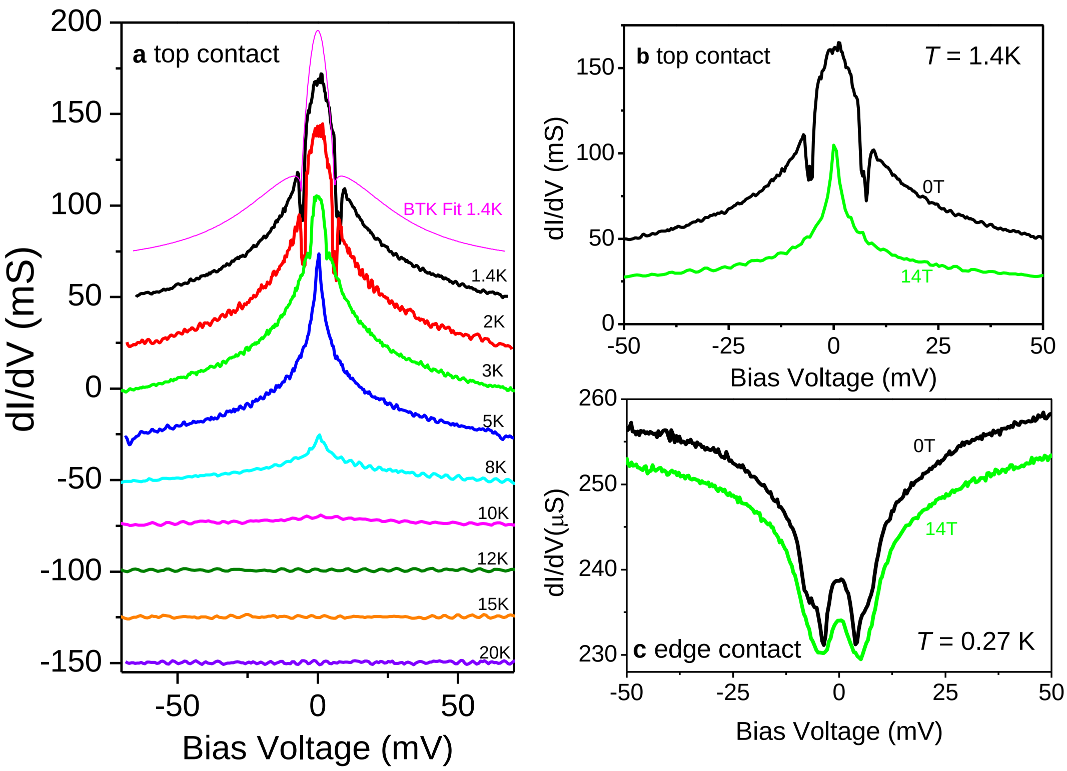

Supplementary Figure 1 | Differential conductance of a low-*Z* point-contact piercing through the Bi_2Te_3 top surface. (**a**) Temperature dependence from 1.4 K to 20 K. Offsets have been added for clarity. The spectra is in the low-Z Andreev limit, thus giving rise to a positive signature due to superconducting Andreev reflections. A fit with a two gap BTK model to the 1.4 K data has been added (with offset for clarity) (**b**) Spectra at 1.4 K in 0 and 14 T showing a complete suppression of $\Delta/_1$, while Δ_2 is hardly affected by the strong field. (**c**) 0 T and 14 T data from the high-*Z* edge contact for comparison (same data but with offset as in Figure 3a of the main article).

Supplementary Discussion

In Supplementary Figure 1 data from a second point contact on the top surface is shown. However, in contrast to Figure 2 in the main article, where a scanning probe was put very gently in contact with the Bi_2T_3 layer carefully avoiding damage to this layer, in the present experiment the scanning probe was pushed with force on the surface thus piercing a hole through the heterostructure, which was optically visible after the end of the experiment. The contact is thus similar to the edge contact and the tunneling current enters the Bi_2Te_3 layer and the interface layer in parallel. The contact has been adjusted to be in the low-Z regime and the spectra are dominated by Andreev reflections, thus showing positive features in contrast to the negative gap-like features in the high-Z tunneling regime. This experiment serves us as a control experiment: By probing Andreev reflections we can identify a superconducting origin of the different gaps in the different temperature regimes.

All data at 12 K or above are absolutely flat, demonstrating the absence of phase coherent Cooper pairs above 12 K. Below 12 K, a sharp peak from Andreev reflections develops, which for 8 and 5 K clearly shows the characteristic shape of a nodal order parameter [10]. In our contact configuration we are injecting the current likely in different in-plane directions so that we observe a superposition of spectra from nodal and anti-nodal directions. This suggests that the ZBCP observed in the data from the edge may have its origin in nodal order parameter symmetry. The edges of the peak extend well beyond 50 meV and thus are attributed to the large superconducting gap Δ_2 observed on the edge contact. At 3 K, two small side peaks become visible and at lower temperature two dips develop

around 6 meV with a broader ZBCP growing out of the center. We attribute the large gap to Δ_2 observed on the edge contact, which is thus likely also responsible for the ZBCP observed at the edge. A magnetic field of up to 14 T (b) has little effect on this gap, while the smaller gap structure that obviously corresponds to Δ_1 becomes strongly suppressed by fields less than 1 T. A similar behavior is seen for the edge contact (Figure 3a of main article), where Δ_2 is very robust to a field up to 14 T, while Δ_1 is suppressed in smaller fields. The broad ZBCP within the central gap below 3 K shows the presence of a large number of in-gap states that cause strong Andreev reflections in our highly transparent contact. The fact that Andreev reflections with signatures of both Δ_1 and Δ_2 are observed confirms that both gaps have a superconducting origin. In the top contact shown in the main article in Figure 2, only Δ_1 was observed on top of the Bi_2Te_3 layer, while Δ_2 appears when the tip pierces through the top layer thus probing both the Bi_2Te_3 layers. This confirms the origin of Δ_1 to be due to superconductivity in the Bi_2Te_3 layer. The fact that it appears below the temperature where zero resistance is established due to the onset of phase coherence at T_{BKT} suggests its origin to be proximity induced superconductivity, although we cannot clearly rule out an intrinsic origin of superconductivity in the top Bi_2Te_3 layer.
